# Hepatocellular carcinoma and AIM2: Therapeutic potential through regulation of autophagy and macrophage polarization

**DOI:** 10.1002/iid3.70002

**Published:** 2024-09-02

**Authors:** Shuangshuang Xie, Cuiyun Wang, Xiaoyan Liu, Cheng Li, Jinhong Yu, Shumin Ma, Qiang Li, Wenjun Du

**Affiliations:** ^1^ Department of liver diseases, Shandong public health clinical center Shandong university Jinan Shandong China

**Keywords:** Absent in Melanoma 2, autophagy, cell proliferation, hepatocellular carcinoma, macrophage polarization

## Abstract

**Objective:**

Hepatocellular carcinoma (HCC) poses a significant challenge to global health. Its pathophysiology involves interconnected processes, including cell proliferation, autophagy, and macrophage polarization. However, the role of Absent in Melanoma 2 (AIM2) in HCC remains elusive.

**Methods:**

The expression of AIM2 in Huh‐7 and Hep3B cell lines was manipulated and cell proliferation, autophagy, apoptosis, and migration/invasion, together with the polarization of M2 macrophages, were evaluated. The markers of autophagy pathway, LC3B, Beclin‐1, and P62, underwent examination through Western blot analysis. An autophagy inhibitor, 3‐MA, was used to measured the role of autophagy in HCC. Finally, the effect of AIM2 overexpression on HCC was further evaluated using a subcutaneous tumor model in nude mice.

**Results:**

Our results established that AIM2 overexpression inhibits HCC cell proliferation, migration, and invasion while promoting apoptosis and autophagy. Conversely, knockdown of AIM2 engendered opposite effects. AIM2 overexpression was correlated with reduced M2 macrophage polarization. The autophagy inhibitor substantiated AIM2's role in autophagy and identified its downstream impact on cell proliferation, migration, invasion, and macrophage polarization. In the in vivo model, overexpression of AIM2 led to the inhibition of HCC tumor growth.

**Conclusion:**

The findings underscore AIM2's crucial function in modulating major biological processes in HCC, pointing to its potential as a therapeutic target. This study inaugurally demonstrated that AIM2 activates autophagy and influences macrophage polarization, playing a role in liver cancer progression.

## Graphical abstract

In liver cancer cells, activation of the AIM2 inflammasome promotes autophagy and inhibits M2‐type polarization of macrophages.

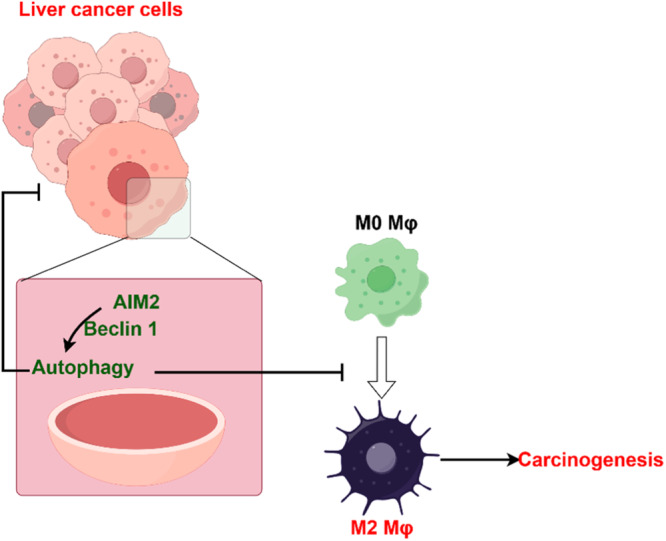

## 1 INTRODUCTION

Hepatocellular carcinoma (HCC) has a high incidence in China, accounting for approximately half of the global HCC cases.^1^ This is mainly due to the high infection rates of hepatitis B and C viruses in the country, which are leading causes of HCC.^2^ Other risk factors include liver cirrhosis, long‐term alcohol consumption, high‐fat diets, and certain genetic factors.^3^ Early symptoms of HCC are not obvious, and most patients are diagnosed in late stages. Surgical resection is the first‐choice treatment for HCC, though is only suitable for early‐stage cases.^4^ For patients ineligible for surgery, other treatments can be used to control tumor growth and alleviate symptoms.^4^ For example, Sorafenib and Lenvatinib are two commonly used targeted drugs for treating advanced HCC.^5^, ^6^ Some immune checkpoint inhibitors, such as PD‐1 and PD‐L1 inhibitors, have shown therapeutic benefits in advanced HCC cases.^7^ Clinicians often develop personalized treatment plans based on individual patients’ conditions, adopting a multidisciplinary approach. However, liver cirrhosis and other impairments in liver function often accompany HCC, limiting the feasibility of surgery and some treatments. Certain HCC patients exhibit drug resistance to targeted drugs and chemotherapy, constraining treatment efficacy and sustained tumor control.^8^, ^9^ Consequently, researchers and healthcare practitioners continue to investigate the underlying mechanisms of HCC to develop novel drugs, aiming to improve survival rates and treatment outcomes for patients.

Given their dual roles, macrophages play a significant role in the immune response within HCC, the most common type of primary liver cancer.^10^ They can exert a direct impact on tumors, such as digesting cancer cells and producing cytokines capable of activating other immune cells.^11^ Nonetheless, HCC cells can suppress the activity of macrophages by producing specific molecules, thereby enabling the tumor to evade immune attack.^12^ In HCC, the polarization state of macrophages influences their role within the immune response. M1 macrophages, activated by bacteria or cellular wall components, can generate an antitumor response. On the other hand, M2 macrophages can be primarily activated by specific factors such as fungal cells, parasites, immune complexes, complements, apoptotic cells, macrophage colony stimulating factor (MCSF), interleukin‐4 (IL‐4), IL‐13, IL‐10, tumor growth factor beta (TGF‐β).^13^ M2 macrophages may facilitate tumor evasion from immune surveillance and promote tumor growth. Therefore, macrophages play a complex and crucial role in the development of HCC.^14^ Their polarization state holds significant relevance to the progression of HCC and the corresponding immune response.^14^

Absent in Melanoma 2 (AIM2) is a critical molecule involved in immune regulation and inflammatory processes.^15^ As an infection sensor, AIM2 recognizes and binds to intracellular DNA, particularly exogenous DNA, released during viral infections or as a result of cell damage.^15^ The complex formed by AIM2 interacting with the adaptor protein ASC (Apoptosis‐associated speck‐like protein containing a CARD) activates Caspase‐1, leading to inflammatory responses.^16^ Beyond its immune regulatory function, study has demonstrated that AIM2 also exerts substantial influence in tumor biology.^17^ AIM2 suppresses tumor growth and metastasis by activating immune cells, such as macrophages.^18^ It promotes the production of IL‐1β and IL‐18, enhances T cell and NK cell activities, and strengthens antitumor immunity.^19^ While progress has been significant in researching AIM2 in tumors, several unanswered questions and challenges remain. While, the regulatory mechanisms of AIM2 and its interactions with other signaling pathways require further investigation. Research has shown that AIM2 can mediate the M1 polarization of macrophages and inhibit M2 polarization, playing a key regulatory role in related diseases. For example, Heinisch O et al. suggest that the activation of the AIM2 inflammasome promotes the inflammation response in macrophages.^20^ The regulation of AIM2 expression can aid in controlling inflammatory responses associated with the involvement of macrophages.^21^ AIM2 has demonstrated downregulated in liver cancer tissues, but enhancing AIM2 expression can inhibit the malignancy of liver cancer cells.^15^ AIM2 can activate the hepatic inflammasome, release inflammatory factors, and induce tumor cell pyroptosis.^22^ The occurrence of this tumor cell pyroptosis may be related to AIM2's regulation of the mTOR‐S6K1 pathway.^23^ In our previous studies, we have discovered a strong correlation between AIM2 expression and hepatic inflammation under Hepatitis B Virus (HBV) infection^24^, ^25^, However, the relationship between AIM2 expression and the microenvironment of hepatocellular carcinoma HCC has not yet been explored.

In this study, we aim to explore the biological role of AIM2 in HCC cells, and investigate its regulatory effects on the M2 polarization of macrophages. By manipulating AIM2 expression in HCC cell lines, we will evaluate cell proliferation, autophagy, apoptosis, and migration/invasion, alongside the polarization state of macrophages. Additionally, we will explore the autophagy pathway and use in vivo models to assess the impact of AIM2 overexpression on tumor growth. Our findings may provide a comprehensive understanding of the mechanisms through which AIM2 influences HCC progression and immune responses within the tumor microenvironment. Ultimately, this research could lay the foundation for new therapeutic strategies targeting AIM2 to improve treatment outcomes for HCC patients.

## 2 MATERIALS AND METHODS

### 2.1 Cell culture

Normal hepatocyte (L‐02) and HCC cells (Huh‐7, Hep3B, and PLC/PRF/5) were gained from the Cell Bank, Chinese Academy of Sciences (Shanghai, China). And L‐02 cells were incubated in DMEM/F12 (Gibco); Huh‐7 and Hep3B cells were cultured in DMEM (Gibco); PLC/PRF/5 cells were grown in MEM (Gibco, cat. no. 41500034). All three media were supplemented with FBS (Gibco, Cat. no.10099‐141). All cells were placed at 37°C with 5% CO_2_.

Macrophages used in this experiment were obtained by inducing THP‐1 cells. The THP‐1 cell line was purchased from the National Infrastructure of Cell Line Resource. The cells were cultured in RPMI 1640 medium containing 10% FBS and 1% penicillin‐streptomycin. THP‐1 cells were treated with 100 ng/mL PMA for 48 h at 37°C in 5% CO_2_, followed by the removal of non‐adherent cells to obtain M0 macrophages. To induce M2 macrophages, IL‐4 (20 ng/mL) and IL‐13 (20 ng/mL) were added to the M0 macrophage culture for 48 h. M2 macrophages were thus obtained. A Transwell culture system was used to coculture HCC cells with macrophages.

### 2.2 Cell transfection and treatment

AIM2 siRNAs (siRNA#1, #2, and #3) and negative control (NC) were synthesized from GenePharma (Shanghai, China). Empty vector and AIM2 overexpression plasmid were purchased from Hanbio (Shanghai, China). Three‐methyladenine (3‐MA) was obtained from Sigma (Shanghai, China). These siRNAs and plasmids were transfected into Huh‐7 and Hep3B cells using Lipofectamine 3000 (Invitrogen) according to the manufacturer's instructions. After 24 h of transfecting with the AIM2 overexpression plasmid, cells were cocultured with or without 3‐MA (10 mM) for 18 h. Cells were harvested for the next experiment or coculture them with M0 macrophages.

### 2.3 qRT‐PCR

TRIzol reagent (Invitrogen) was adopted to extract total RNA, and the purity of total RNA was identified by an ultraviolet spectrophotometer. reverse transcription was conducted with a reverse transcription kit (Promega). Gene expression was measured through RT‐PCR with the SYBR Premix Ex TaqTM kit (Takara). Relative gene expression was calculated using the 2^−ΔΔCt^ method.

### 2.4 Western blot

Total protein was extracted from the processed Huh‐7 and Hep3B cells using RIPA buffer with a proteinase cocktail. Protein concentration was tested using the BCA kit (Beyotime, China). Protein was denatured, separated in 10% SDS‐PAGE by electrophoresis, and transferred to PVDF membranes (Millipore). The primary antibodies include anti‐AIM2(Abcam), anti‐Caspase 1 (Abcam), anti‐LC3B (Abcam), anti‐Beclin 1 (Abcam), anti‐P62 (Abcam), anti‐Arg‐1 (Abcam), anti‐YM1 (Abcam), anti‐GAPDH (Abcam) were diluted according to the manual. After blocking with 5% skim milk powder, the membranes were immersed in diluted primary antibody overnight at 4°C, followed by secondary antibody (1:1000, Abcam) for 1 h at 37°C. After washing, each sample was addressed with ECL solution (Pierce) and the signals were obtained using a chemiluminescence system (Bio‐Rad, USA).

### 2.5 ELISA

Cell or tissue supernatants were collected, and the levels of TGF‐β, IL‐18, and IL‐1β were confirmed using the ELISA kits (R&D Systems, Inc.) based on the manufacturer's instructions.

### 2.6 Flow cytometry

Macrophages were characterized by flow cytometry for expression of CD68 and CD163. CD68 + CD163+ to label human M2 macrophages.^26^ Cells were incubated with rabbit CD68 (Servicebio, Wuhan, China) and CD163 (Servicebio, Wuhan, China) at a 1:500 dilution for 1 h on ice, followed by a wash with PBS, and subsequent incubation with Fluorescence‐conjugated antibodies for 30 min on ice. After washing with PBS, cells were analyzed by flow cytometry to detect CD68 and CD163 expression levels.

### 2.7 Apoptosis flow cytometry assay

Each group of cells was made into a single‐cell suspension. After washing with PBS, cells were counted at 1 × 10^5^ cells/ml and resuspended. Then the cell suspensions were mixed with 195 μL Annexin V and 5 μL FITC for 15 min at 4°C, and incubated with 5 μL PI solution for 5 min at 4°C. Each group of cells was subjected to flow cytometry (BD Biosciences) detection and analysis.

### 2.8 EdU staining

Cells were seeded in 96‐well plates and incubated with 50 mmol/L EdU for 48 h. Subsequently, the cells were fixed with 4% paraformaldehyde for 15 min and addressed with glycine for 10 min. After DAPI nuclear staining, the Edu‐positive cells were observed and photographed using a fluorescence microscope.

### 2.9 Transwell assay

Each group of Huh‐7 and Hep3B cells was first serum‐starved for 24 h. The upper chamber of Transwell (8 μm pore size, Costar) was added with 200 μl of 1 × 10^4^ cell suspension, and the bottom chamber was supplemented with medium including 10% FBS as a chemoattractant. After incubation for 48 h, cells in the upper chambers were cleared and the migrated or invaded cells were fixed using methanol, and stained using 0.1% crystal violet. The results were photographed with a microscope (Olympus, Japan). Transwell was coated without or with Matrigel (BD Biosciences, USA) for migration and invasion assays.

### 2.10 Co‐IP assay

The treated cells were lysed by RIPA buffer (Beyotime, China) and proteins were extracted. Each sample was added with 1 μg of AIM2 and IgG antibodies (Abcam) overnight at 4°C. Subsequently, the sample was added with the pretreated beads (10 μL) at 4°C for 2‐4 h. After the immunoprecipitation reaction, the samples were centrifuged at 12,000 r/min for 30 s at 4°C. Then the supernatant was removed and agarose beads were washed with 1 mL lysis solution. The above samples were subjected to western blot analysis.

### 2.11 Immunofluorescence (IF) staining

The treated cells were collected, washed, fixed with 4% paraformaldehyde for 30 min, and added with 0.5% TritonX‐100 for 15 min. After closure with 10% goat serum, the cells were treated with LC3B antibody (1:100, Abcam) overnight at 4°C. The following day, the cells were washed with PBS and incubated with fluorescent secondary antibody (Abcam) for 1 h. After DAPI staining for the avoidance of light, the samples were sealed with neutral gum, and the staining of cells was observed under a fluorescence microscope.

### 2.12 Xenografts in mice

Fifteen BALB/C nude mice (4–6 weeks old, 18–22 g) were randomly divided into blank, NC, and AIM2 overexpression groups, with 5 mice in each group. All mice were fed under SPF‐grade conditions. The Huh‐7 cells were stably transfected with AIM2 overexpression plasmid and vector (NC). Each nude mouse was subcutaneously injected with 100 μL of transfected Huh‐7 cells (~2 × 10^6^ cells) in the right axilla. The nude mice and tumors were observed daily. The long and short diameters of the tumors were recorded every 7 days, and the volume of the tumors was calculated. After 4 weeks, the mice were euthanized and the weight of the tumor was measured.

### 2.13 H&E staining

The tumor tissues were fixed in 4% paraformaldehyde for 24 h, routinely paraffin‐embedded, and 4‐μm‐thick sections were prepared. The sections were treated with xylene, and gradient alcohol, stained with hematoxylin for 5 min, washed with water for 1 min, returned to blue with ammonia, and stained with eosin for 2 min. After alcohol dehydration, xylene transparency, and sealing, the sections were observed under a microscope (Olympus, Japan).

### 2.14 TUNEL staining

The sections were dewaxed to water (xylene I for 5 min, xylene II for 5 min, and gradient ethanol) and dripped with 20 μg/ml proteinase K at 37°C for 20 min. After washing, the sections were dripped with TUNEL solution at 37°C for 60 min and DAPI for 10 min. Subsequently, the sections were dehydrated with gradient ethanol, transparent with xylene, and sealed with an anti‐fluorescence quenching agent. Then the staining was observed by fluorescence microscopy.

### 2.15 IHC staining

After paraffin sections were deparaffinized to water, antigen repair was performed with sodium citrate (10 mM, 0.05% Tween 20, pH 6.0) was performed at 95–100°C for 15 min. After washing, the sections were addressed with H_2_O_2_ for 15 min. Subsequently, the sections were blocked with normal goat serum for 30 min, followed by the addition of primary antibody Ki67 (Abcam) overnight at 4°C and secondary antibody (Abcam) at 37°C for 40 min. The sections were reacted with 0.05% diaminobenzidine (DAB) for 5 min, washed with distilled water, counterstained with hematoxylin, and observed with a microscope (Olympus, Japan).

### 2.16 Statistical analysis

The measures were denoted as mean ± SD. SPSS25.0 software (IBM, SPSS, Chicago, IL, USA) was applied for statistical analysis. One‐way ANOVA, followed by Tukey's test, was applied for comparison between multiple groups, and the difference was considered statistically significant at *p* < .05.

## 3 RESULTS

### 3.1 AIM2 is overexpressed or silenced in HCC cells

**Figure 1:**
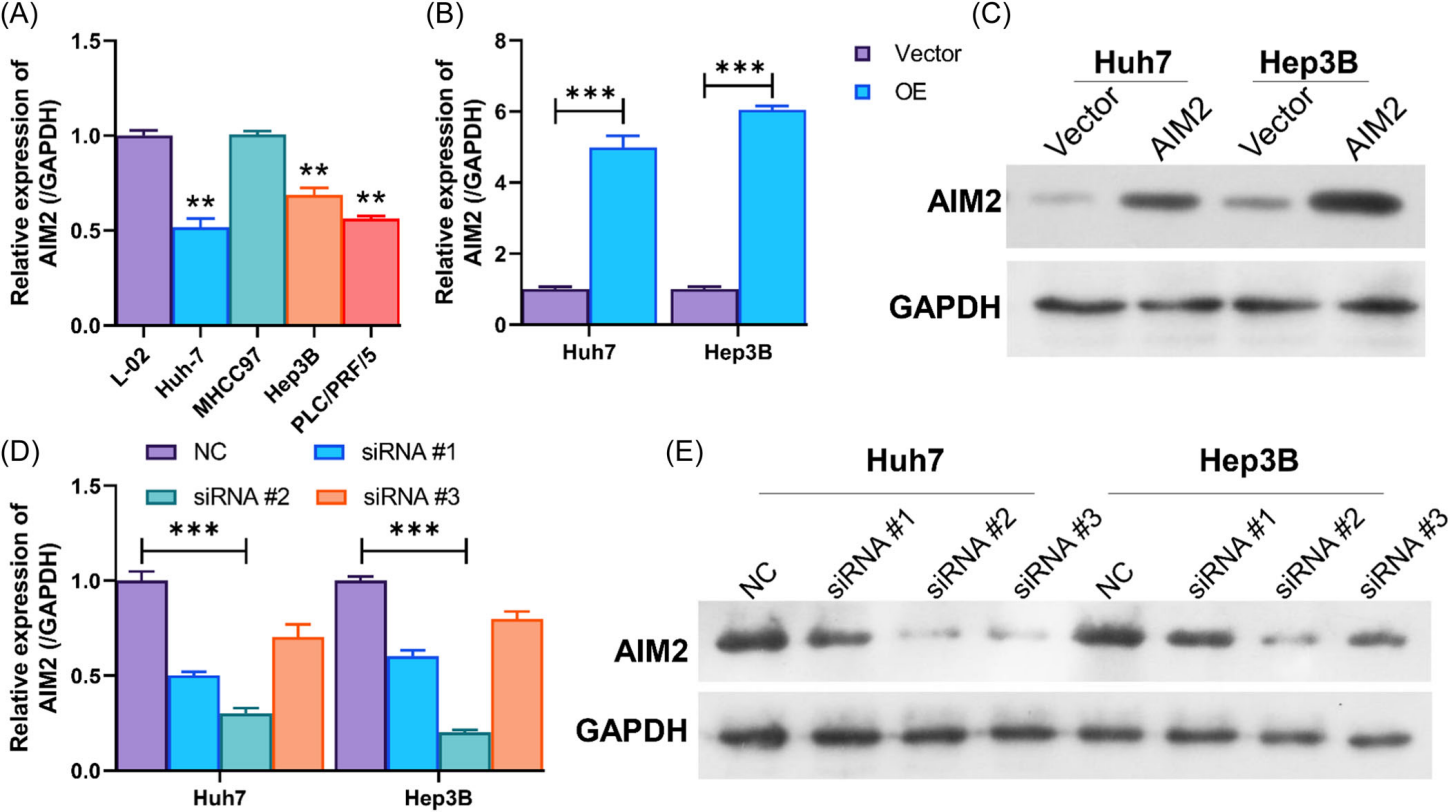
Overexpression and knockdown of AIM2 in HCC cells. (A) AIM2 expression was assessed by qRT‐PCR in normal hepatocytes (L‐02) and HCC cells (Huh‐7, MHCC97, Hep3B, and PLC/PRF/5). (B, C) qRT‐PCR and western blot analysis of AIM2 expression in AIM2‐overexpressed Huh‐7 and Hep3B cells. (D) After transfection with AIM2 siRNAs, AIM2 expression was monitored with qRT‐PCR. (E) The protein level of AIM2 was determined using a western blot in Huh‐7 and Hep3B cells treated with AIM2 siRNAs. **p < .01, ***p < .001.

To study the effect of AIM2 in HCC, AIM2 expression was examined in normal hepatocytes (L‐02) and different HCC cell lines. As displayed in Figure 1A, AIM2 expression was significantly lower in HCC cells (Huh‐7, Hep3B, and PLC/PRF/5) than in the L‐02 cells. While, there is no significant difference in AIM2 expression between MHCC97 and L‐02. Subsequently, AIM2 was overexpressed in Huh‐7 and Hep3B cells, confirmed by qRT‐PCR and western blot (Figure 1B, C). Meanwhile, siRNA targeting AIM2 was used to knockdown AIM2 in Huh‐7 and Hep3B cells. As shown in Figure 1D, E, qRT‐PCR and Western blot showed that siRNAs, including siRNA#1, siRNA#2, siRNA#3, suppressed expression of AIM2 in Huh‐7 and Hep3B cells. Of the siRNAs, #2 exhibited the highest knockdown efficiency in Huh‐7 and Hep3B cells. Therefore, siRNA#2 was used for the subsequent experiments.

### 3.2 AIM2 prevents the progress of HCC

**Figure 2:**
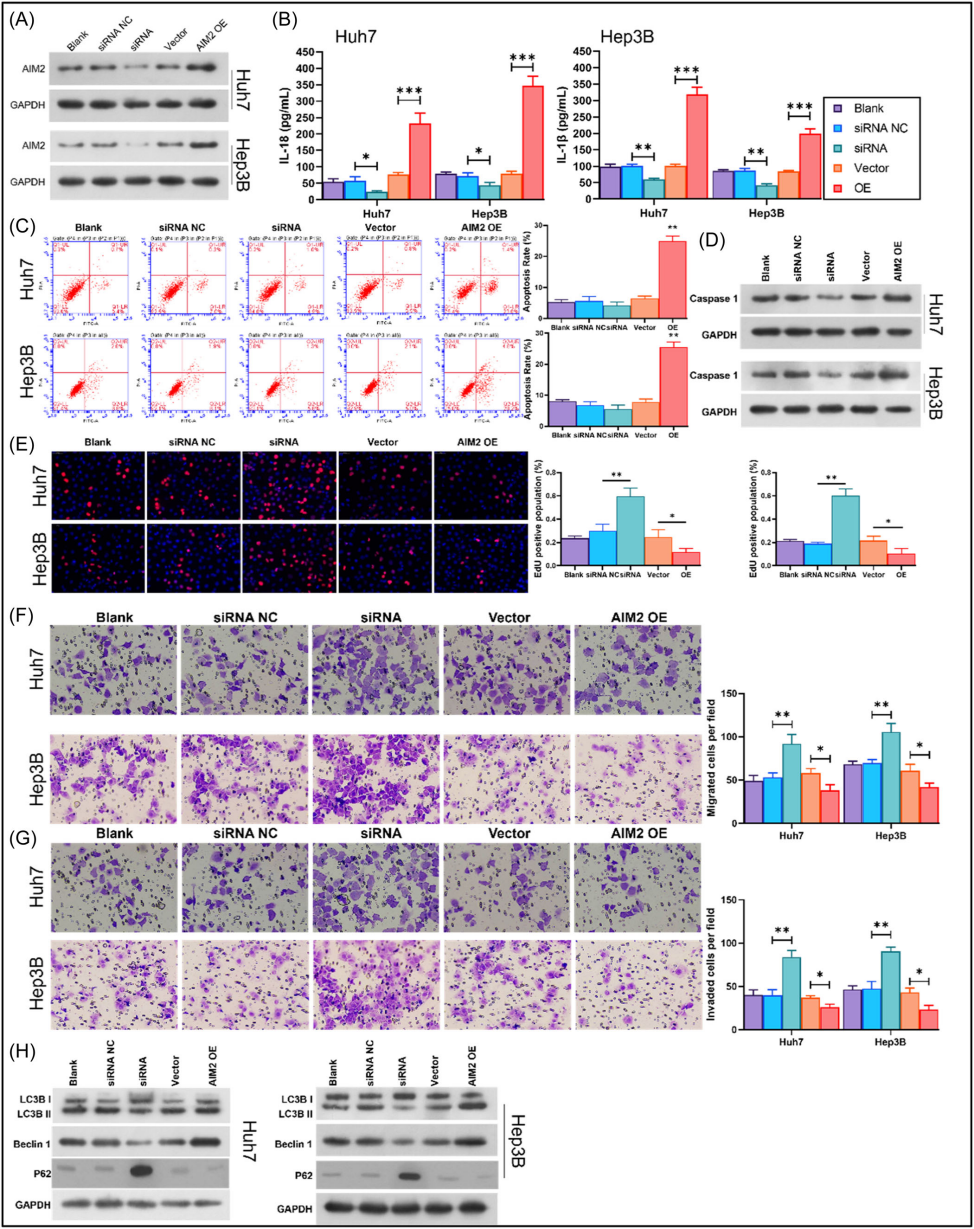
The impacts of AIM2 on the inflammasome, apoptosis, proliferation, autophagy, migration, and invasion of HCC cells. Huh‐7 and Hep3B cells were transfected with AIM2 siRNAs or AIM2 overexpression plasmid, respectively. (A) Expression of AIM2 was evaluated through western blot analysis analysis. (B) IL‐18 and IL‐1β levels were identified using ELISA in the cell supernatant of each group. (C) Flow cytometry was adopted to examine the changes in apoptosis rate. (D) Cleaved Caspase 1 was detected using Western blot analysis analysis. (E) EdU‐positive cells from each group were tested using Edu staining. Magnification, 200×. (F, G) A Transwell assay was conducted to monitor the alterations in cell migration and invasion capacity. Magnification, 200×. (H) LC3B Beclin 1 and P62 levels were confirmed by western blot. *p < .05, **p < .01, ***p < .001.

For functional experiments, western blot experiments were first applied to verify the silencing and overexpression of AIM2 in Huh‐7 and Hep3B cells. After AIM2 was overexpressed or knockdown in Huh‐7 and Hep3B cells (confirmed by Western blot, Figure 2A), IL‐1β and IL‐18 was measured. ELISA results denoted that AIM2 knockdown reduced IL‐18 and IL‐1β levels and AIM2 overexpression elevated IL‐18 and IL‐1β levels in Huh‐7 and Hep3B cells (Figure 2B). Flow cytometry analysis indicated that overexpression of AIM2 increased the apoptosis of Huh‐7 and Hep3B cells significantly (Figure 2C), along with elevated expression of cleaved Caspase 1 (Figure 2D). Besides, EdU staining results showed that AIM2 knockdown increased EdU‐positive cells and AIM2 overexpression decreased EdU‐positive cells (Figure 2E). Transwell assay results, as shown in Figure 2F proved that AIM2 knockdown enhanced cell migration and invasion, while AIM2 overexpression attenuated migration and invasion in Huh‐7 and Hep3B cells (Figure 2F, G). Further, our data also revealed that AIM2 knockdown led to a decrease of autophagy, characterized by suppressed LC3B II (not including LC3B I) and Beclin 1 and elevated P62 in Huh‐7 and Hep3B cells (Figure 2H). These results suggested that AIM2 inflammasome regulated autophagy potentially, to suppress malignant behavior in liver cancer cells.

### 3.3 AIM2 inhibits M2 macrophage polarization

**Figure 3:**
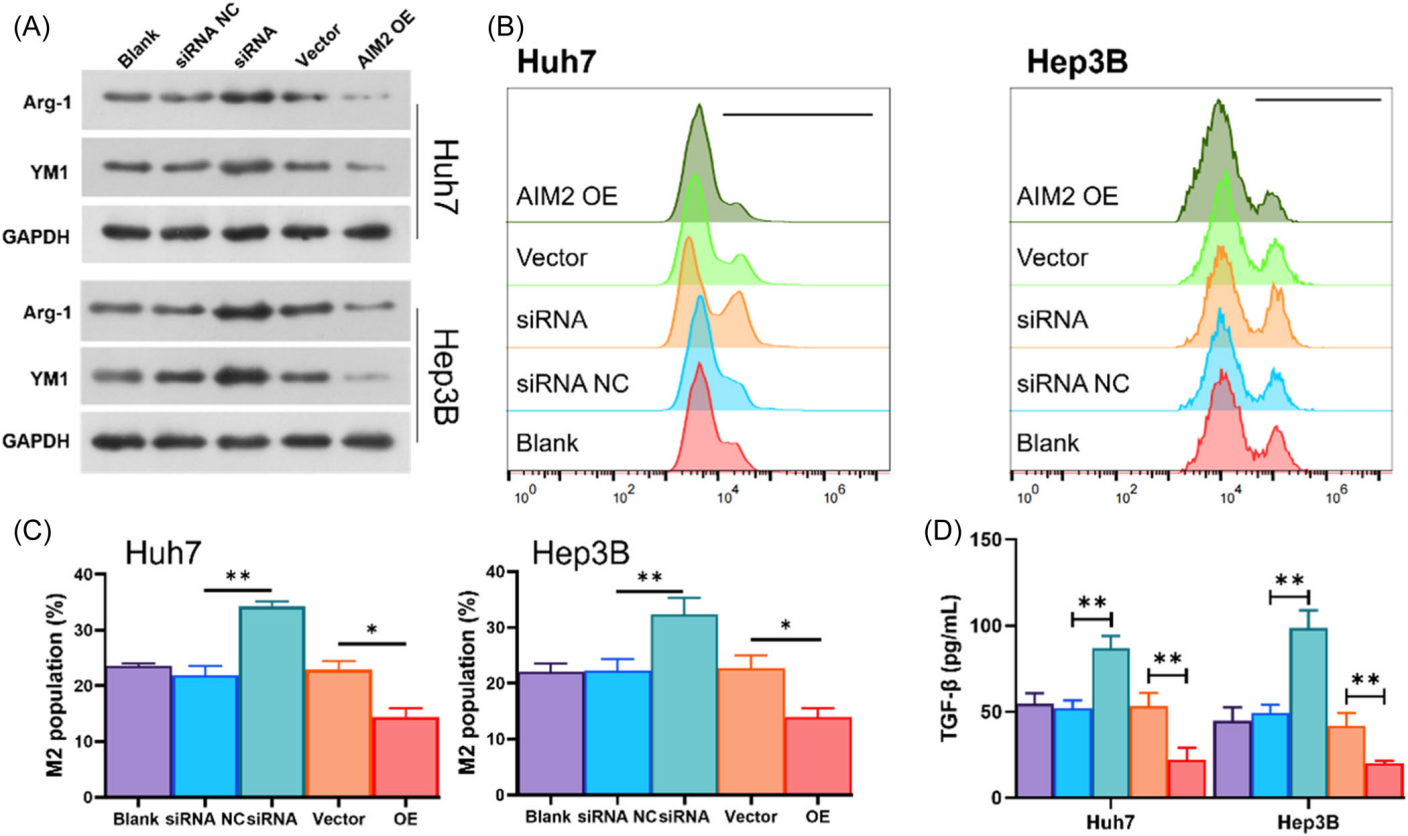
The influence of AIM2 on M2 macrophage polarization. M0 macrophages were cocultured with AIM2 knockdown or overexpressed Huh‐7 and Hep3B cells. (A) M2 macrophage markers (Arg‐1 and YM1) were assessed by Western blot. (B, C) M2 macrophages (CD68+/CD163+) was quantified using flow cytometry. (D) ELISA analysis of TGF‐β level affected by AIM2 knockdown or AIM2 overexpression in coculture system supernatant. *p < .05, **p < .01.

To further clarify the influence of expression of AIM2 in HCC cells on macrophage polarization, HCC cells were cocultured with M0 macrophages. As presented in Figure 3A, M2 macrophages biomarkers, Arg‐1 and YM1 expressions in macrophages were elevated when cocultured with HCC cells treated with siRNA. While, a reduction of Arg‐1 and YM1 was observed in macrophages when cocultured with AIM2‐overexpressed HCC cells (Figure 3A). Afterwards, macrophages were labeling with CD68, and cells with CD68 + /CD163+ was regarded as M2 macrophages. As results shown in Figure 3B, C, the percentage of M2 macrophages was elevated when cocultured with AIM2 knockdown HCC cells, with an elevated TGF‐β level, and the percentage of M2 macrophages was decreased significantly when cocultured with AIM2‐overexpressed HCC cells, along with a suppressed TGF‐β level (Figure 3B–D).

### 3.4 AIM2 promotes inflammasomes, and apoptosis, and prevents proliferation by the activating autophagy

**Figure 4:**
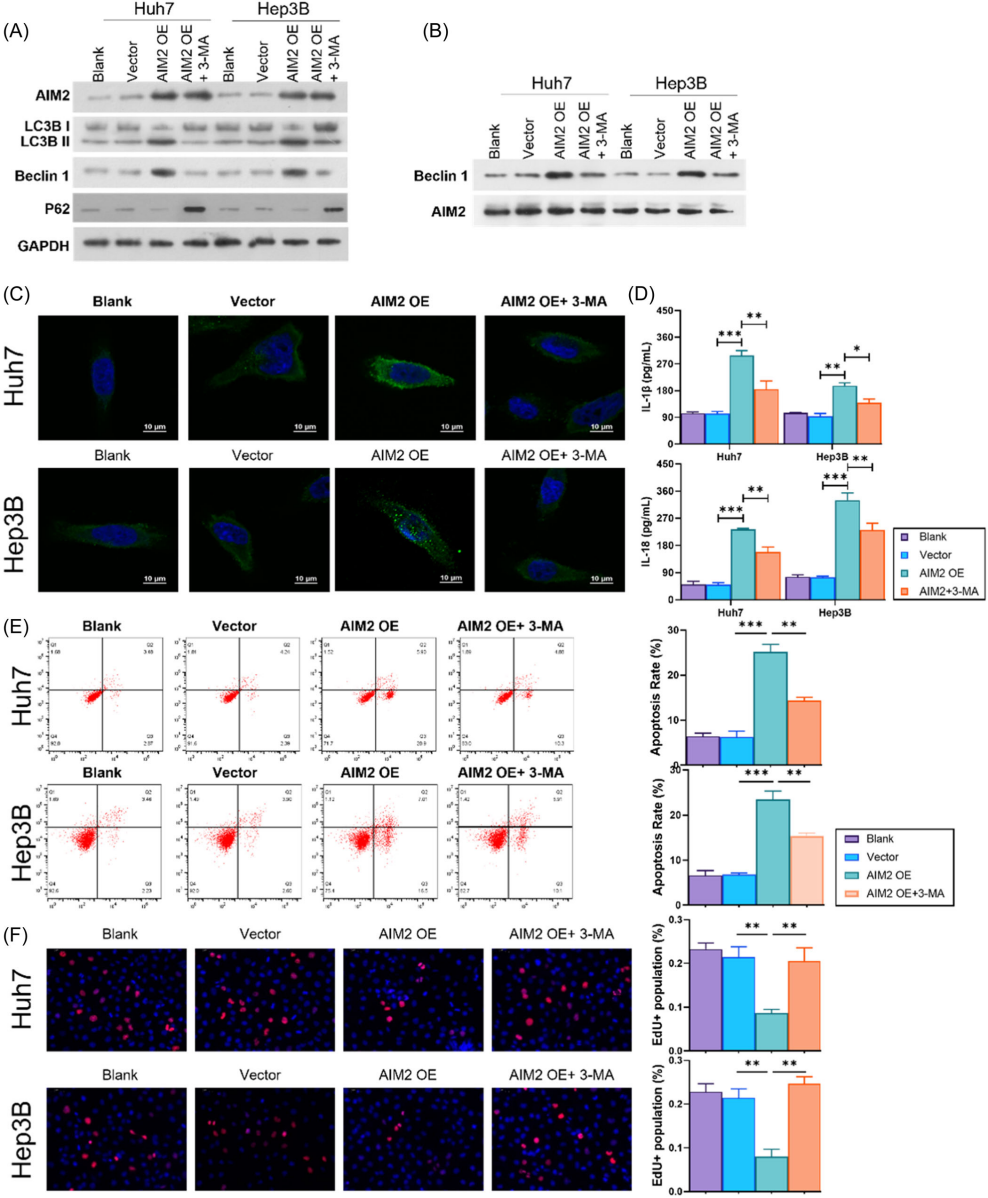
3‐MA abolishes the AIM2 overexpression‐inhibited cellular processes in Huh‐7 and Hep3B cells. An autophagy inhibitor, 3‐MA, was adopted to treat AIM2‐overexpressed Huh‐7 and Hep3B cells. (A) AIM2, LC3B, Beclin 1 and P62 expression was measured in HCC cells using western blot analysis. (B) Co‐IP assay was performed to monitor the binding of AIM2 to Beclin 1. (C) Autophagy was observed using IF in HCC cells. Magnification, 400×, scale bar = 10 μm. (D) IL‐18 and IL‐1β levels enhanced by AIM2 overexpression was reversed by 3‐MA. (E) Cell apoptosis of HCC cells was measured using flow cytometer. (F) Cell proliferation was detected using EdU staining and quantified by EdU‐positive cell percentage. Magnification, 200×. *p < .05, **p < .01, ***p < .001.

To explore the role of autophagy in HCC‐Macrophage axis, an autophagy inhibitor, 3‐MA, was involved in HCC cells. As shown in Figure 4A, the LC3B II and beclin 1 expression was suppressed, while P62 was increased in AIM2‐overexpressed HCC cells after treated with 3‐MA (Figure 4A). Co‐IP experiment disclosed that overexpression of AIM2 enhanced the enrichment of Beclin 1 in Huh‐7 and Hep3B cells, suggesting an endogenous binding between the two proteins (Figure 4B). IF assay results further showed that AIM2 enhanced LC3B expression in the Huh‐7 and Hep3B cells, and 3‐MA attenuated the upregulation of LC3B in AIM2‐overexpressed HCC cells (Figure 4C). Results found that the elevated IL‐18 and IL‐1β by AIM2 overexpression was partially reversed by 3‐MA in culture medium (Figure 4D). While, the apoptosis rate enhanced by AIM2 overexpression was recovered from 3‐MA treatment (Figure 4E), along with a reversed EdU‐positive cells (Figure 4F). These results suggested that autophagy played a crucial role in the process of the AIM2 inflammasome suppressing malignant cell behaviors.

### 3.5 Suppression of M2 macrophage polarization reversed in macrophages cocultured with 3‐MA treated in AIM2‐overexpressed HCC cells

**Figure 5:**
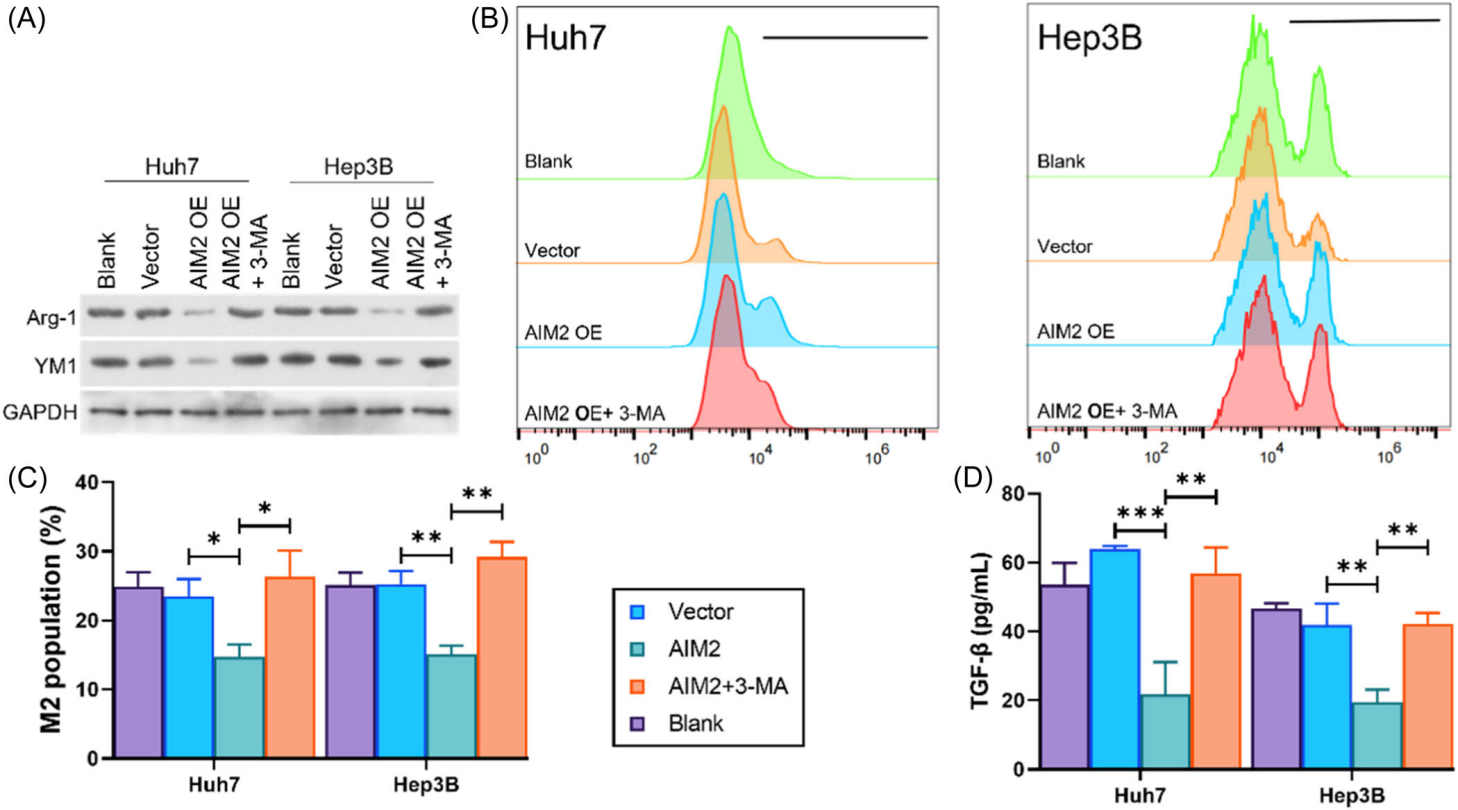
3‐MA treated on HCC cells reversed the tendency of M2 macrophage polarization by AIM2 overexpression. (A) Arg‐1 and YM1 expression was measured in macrophages after cocultured with HCC cells. (B, C) the percentage of CD68+/CD163+ was measured using Flow cytometry. (D) ELISA kit was applied to detected the level of TGF‐β in cellular supernatant. *p < .05, **p < .01, ***p < .001.

To confirm the effect of autophagy in HCC cells on M2 macrophage polarization, macrophages were cocultured with 3‐MA treated AIM2‐overexpressed HCC cells. Results showed that weakened Arg‐1 and YM1 expression, suppressed by cocultured with AIM2‐overexpressed HCC cells, was reversed by 3‐MA (Figure 5A). Additionally, results also presented that the percentage of CD68 + /CD163+ macrophages was increased after cocultured with 3‐MA treated AIM2‐overexpressed HCC cells (Figure 5B, C), Meanwhile, the inhibitory effect of AIM2 overexpression on TGF‐β level was partially offset by 3‐MA in coculture system (Figure 5D).

### 3.6 Overexpression of AIM2 suppresses HCC cell growth in vivo

**Figure 6:**
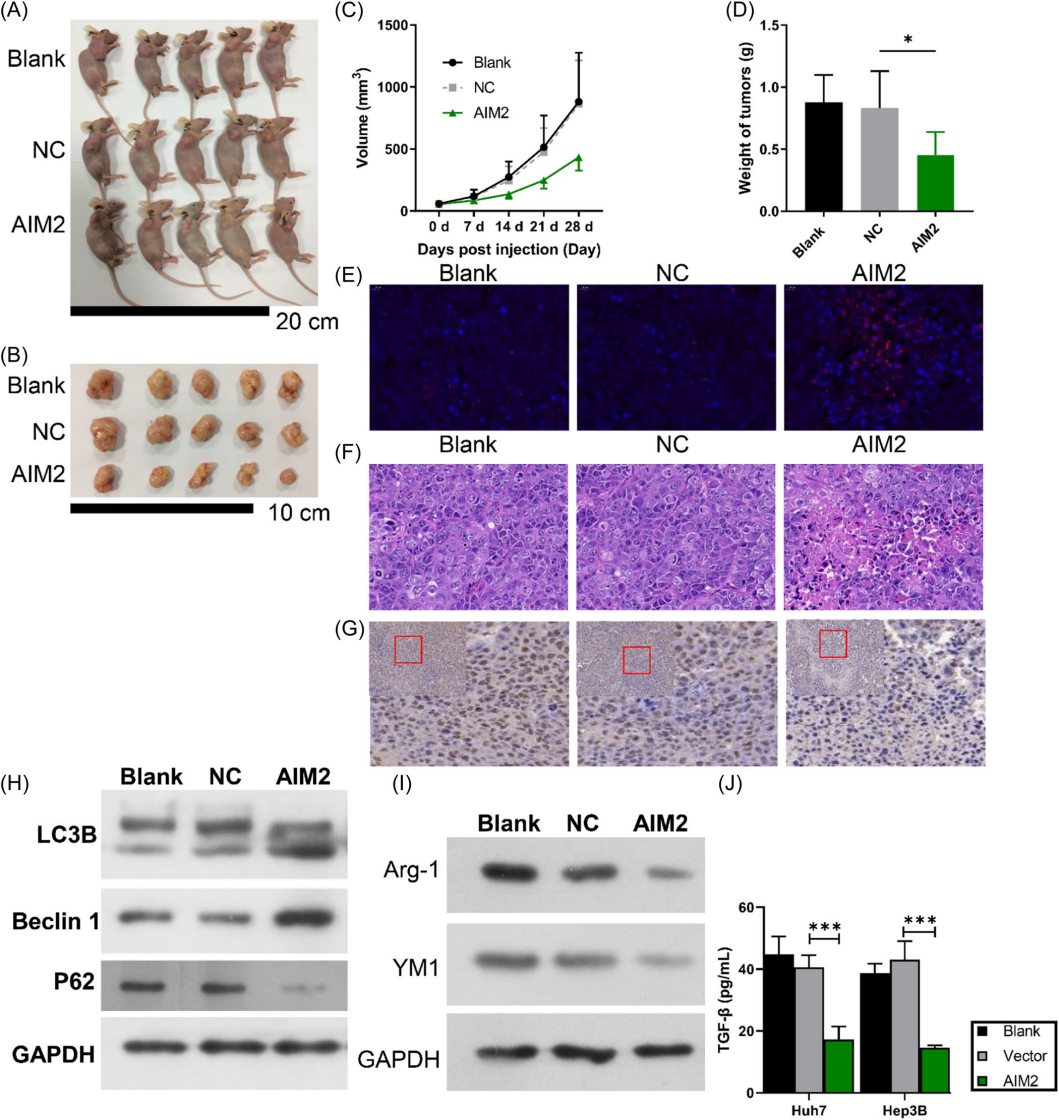
AIM2 overexpression prevents tumor growth in vivo. AIM2‐overexpressed Huh‐7 cells were subcutaneously injected into the nude mice for 28 days. (A) Images of nude mice in blank, NC, and AIM2 overexpression groups. (B) Images of tumor tissues in each group. (C) The tumor volume was calculated every seven days. (D) The tumor weight was weighed at 28 days. (E) TUNEL staining exhibited apoptotic cells in tumor tissues. Magnification, 200×. (F) The histological morphology of tumors was assessed using H&E staining. Magnification, 200×. (G) IHC staining of Ki67 in the tumor tissues. Magnification, 100×. (H) Expression of LC3B Beclin1 and P62 was determined by western blot in tumor tissues. (I) Western blot analysis results of Arg‐1 and YM1 expressions in tumor tissues. (J) TGF‐β level was measured using ELISA kit. *p < .05, ***p < .001.

To clarify the role of AIM2 in liver cancer onset and progression, this study conducted experiments based on xenograft model. The results displayed that the volume and weight of tumors was suppressed by AIM2 overexpression (Figure 6A–D). Moreover, TUNEL assay results showed that apoptotic cells, labeled in red, was distributed more widely compared to tumor from NC (Figure 6E). H&E staining results showed extensive nuclear condensation and cytoplasmic rarefaction in tumor tissues from the AIM2 overexpression group (Figure 6F), along with a decreased Ki67 expression (Figure 6G). Western blot denoted that AIM2 overexpression resulted in an elevation in the expression of LC3B II and Beclin1, and suppressed P62 in tumor tissues (Figure 6H). Besides, AIM2 overexpression also could downregulate expression of M2 markers (Arg‐1 and YM1) in the tumor tissues (Figure 6I). Accordantly, the level of TGF‐β presented an suppressive tendency in tissues from mice of AIM2 overexpression (Figure 6J). These data confirmed the inhibitory effect of AIM2 overexpression on HCC tumor growth.

## 4 DISCUSSION

HCC is among those cancers characterized by early stealthiness and high malignancy. In China, both the incidence rate of liver cancer and the number of fatalities caused by the disease continue to rise annually. Despite significant strides made in basic liver cancer research over recent years, the treatment outcomes for patients afflicted with advanced stages of this disease still need to be improved.^1^ In this study, we embarked upon investigations within a microenvironment modeled by liver cancer cells cocultured with macrophages. The impact of AIM2 on the malignant biological behaviors of liver cancer cells in vitro, and studied the effect of liver cancer cells on the M2 polarization of macrophages were investigated. Moreover, we probed into the role played by autophagy in the M2 polarization process of macrophages. Our primary goal was to illuminate the distinctive features of the tumor microenvironment during the onset and progression of HCC.

The innate immune system is composed of a suite of pattern recognition receptors (PRRs) that serve as barriers through recognizing pathogen‐associated molecular patterns and damage‐associated molecular patterns.^27^ Among PRRs, AIM2 specifically identifies double‐stranded DNA (dsDNA) either associated with pathogens or originating from the host cytoplasm. AIM2 recruits ASC (apoptosis‐associated speck‐like protein containing a CARD) and triggers the assembly of Caspase‐1‐dependent inflammasomes, culminating in the maturation of IL‐18 and IL‐1β, which initiates an innate immune response against pathogen invasion.^28^ Research has demonstrated that the AIM2 inflammasome not only plays a protective role in infectious diseases but also serves as a pivotal regulator in noninfectious conditions, such as cancer.^29^ Research indicates that the AIM2‐involved inflammasome plays a key regulatory role in the development and progression of liver cancer. Research by Claudia Martínez‐Cardona et al. reveals that AIM2 exhibits aberrant expression in diethylnitrosamine‐induced animal model of liver cancer, accompanied by heightened inflammasome activity. Additionally, there is an increase in the secretion of the inflammatory cytokine IL‐1β.^29^ Ma et al. also confirmed that overexpression of AIM2 in liver cancer cells can suppress the activity of the mTOR/S6K1 pathway, thereby suppress malignant behaviors such as liver cancer cell proliferation and metastasis. However, when AIM2 is inhibited, the activity of the mTOR/S6K1 pathway is enhanced, promoting the development of liver cancer.^23^ AIM2 displayed antitumor activity in tumor cells, and within HCC cells, the expression of Caspase‐1 and the concentrations of IL‐1β and IL‐18 were positively correlated with AIM2 expression. Thus, HCC cells might modulate the polarization of macrophages through secretion of inflammatory cytokines and other substances.

Macrophages play a crucial modulatory role in the emergence and advancement of HCC. This regulatory function is primarily realized through polarization into pro‐inflammatory or anti‐inflammatory types to reshape the tissue microenvironment.^30^ A clinical study has revealed that effective HCC medications can reduce the distribution of M2 macrophages in the tissues of liver cancer patients, thereby enhancing the infiltration of M1 macrophages.^31^ Tumor‐associated macrophages (TAMs), a subtype of macrophages residing within tumor microenvironments, are considered instrumental in bridging the gap between inflammation and cancer. They often display distinct physiological features compared to other macrophages in the body. Research confirms that the AIM2 inflammasome plays a pivotal regulatory role in macrophage polarization. Activation of the AIM2 inflammasome in macrophages and the subsequent production of IL‐1β contribute significantly to cytokine release syndrome in cancer patients undergoing CAR‐T therapy, and limiting the AIM2 pathway potentially mitigates adverse survival outcomes following CAR‐T treatment, thus implicating a connection between AIM2 and the promotion of pro‐inflammatory macrophage polarization.^32^ Additionally, AIM2 promotes the polarization shift from anti‐inflammatory M2 TAMs towards pro‐inflammatory M1 phenotype through inflammasome signaling activation, thereby enhancing tumor‐killing activity.^33^ Interestingly, reports suggest that AIM2 can activate polarization toward M2 macrophages, contributing to immune escape in lung cancer therapy.^34^ This discrepancy may be related to the differing roles inflammation plays within tumors. However, no studies have yet explored the relationship between AIM2 and macrophage polarization in HCC. In this study, we found that the upregulation of AIM2 effectively inhibits the malignancy of liver cancer cells, whereas its downregulation amplifies malignant characteristics of liver cancer cells. Further in vitro experiments showed that an overexpression of AIM2 in liver cancer cells favors the inhibition of M2 macrophage polarization. This finding implies that the expression levels of AIM2 in HCC cells directly mediate the polarization of macrophages and the formation of TAMs.

Autophagy is an intracellular self‐degradation process, through which a cell can degrade and recycle its damaged or outdated structures, maintaining conditions essential for life activities. This mechanism significantly impacts various physiological processes, such as alleviating cellular stress, fine‐tuning energy homeostasis, and countering cellular senescence and pathogenesis. The relationship between macrophage polarization and the occurrence of autophagy is complex. In recent report, Lv et al. find a positive correlation linking autophagy with the M1 polarization state of macrophages.^35^ Contrarily, research spearheaded by Hu et al. reveals that the suppressing of autophagy leads to an increase in M1 polarization within macrophages.^36^ However, Zhu et al have found that when autophagy in tumor cells is suppressed, macrophages tend to shift towards M2 polarization, pushing the microenvironment towards a direction that facilitates tumor immune tolerance.^37^ Tian et al., suggests that the PI3K/AKT/mTOR pathway can abate inflammatory reactions in lung.^38^ While, researchers also have found that AIM2 suppresses tumor cell malignant biological properties by inactivating PI3K/AKT/mTOR pathway in osteosarcoma cells.^39^ Yet another study finds that the activation of the PI3K/AKT/mTOR pathway can significantly improve M1 polarization of macrophages and their associated inflammatory responses.^40^ Thus, AIM2 may promote autophagy through PI3K/AKT/mTOR pathway in HCC. Research indicates that activation of the AIM2/caspase‐1 inflammasome can promote autophagy and apoptosis in HCC cells, potentially exerting an inhibitory effect on liver cancer progression.^41^ In this study, we found that overexpression of AIM2 in liver cancer cells exerts an inhibitory effect on M2 phenotype macrophage polarization. Additionally, the experiments revealed that inhibiting autophagy in macrophages restored their inclination towards M2 polarization. Future research should focus on exploring the underlying mechanisms of macrophage autophagy in the context of coculturing with liver cancer cells. Additionally, it is important to investigate how AIM2 triggers the modulation of macrophage polarization induced by liver cancer cells. NF‐κB has been proven to play a key role in macrophage polarization, and studies have demonstrated that NF‐κB can activate the AIM2 inflammasome.^42^, ^43^ How to induce the intracellular AIM2 inflammasome is also a topic worth further investigation.

In conclusion, this study was designed to explore the regulatory role of AIM2 expression in liver cancer cells. This study suggested that AIM2 inflammasome affected M2 macrophages polarization within the tumor microenvironment. We also scrutinized the influence of autophagy on the M2 polarization of macrophages. Through these efforts, we have initially elucidated the mechanisms connecting AIM2 to the onset and progression of liver cancer, thereby offering valuable insights and a groundwork for future clinical treatments.

## AUTHOR CONTRIBUTIONS

**Shuangshuang Xie**: Data curation; Formal analysis; Investigation; Methodology; Software; Validation; Visualization; Writing—original draft. **Cuiyun Wang**: Investigation; Methodology; Software; Supervision; Validation; Visualization. **Xiaoyan Liu**: Data curation; Formal analysis; Resources; Software; Visualization. **Cheng Li**: Data curation; Investigation; Methodology; Validation; Visualization. **Jinhong Yu**: Data curation; Formal analysis; Visualization. **Shumin Ma**: Data curation; Methodology; Software; Visualization. **Qiang Li**: Formal analysis; Investigation; Methodology; Software; Validation. **Wenjun Du**: Conceptualization; Funding acquisition; Project administration; Writing—review and editing.

## CONFLICT OF INTEREST STATEMENT

The authors declare no conflicts of interest.

## ETHICS STATEMENT

The animal experimental protocol was reviewed and approved by the Experimental Animal Ethics Committee of Shandong public health clinical center (Jinan, China).

## Data Availability

All data generated or analyzed during this study are included in this published article.
